# “You can’t chain a dog to a porch”: a multisite qualitative analysis of youth narratives of parental approaches to substance use

**DOI:** 10.1186/s12954-019-0297-3

**Published:** 2019-04-05

**Authors:** Allie Slemon, Emily K. Jenkins, Rebecca J. Haines-Saah, Zachary Daly, Sunny Jiao

**Affiliations:** 1 School of Nursing, University of British Columbia, T201-2211 Westbrook Mall, Vancouver, BC V6T 2B5 Canada; 2 Community Health Sciences, University of Calgary, 2500 University Drive NW, Calgary, AB T2N 1N4 Canada

**Keywords:** Youth, Family, Harm reduction, Substance use, Zero-tolerance, Abstinence, Risk environment, Multisite qualitative research

## Abstract

**Background:**

Reducing harms of youth substance use is a global priority, with parents identified as a key target for efforts to mitigate these harms. Much of the research informing parental responses to youth substance use are grounded in abstinence and critiqued as ineffective and unresponsive to youth contexts. Parental provision of substances, particularly alcohol, is a widely used approach, which some parents adopt in an attempt to minimize substance use harms; however, research indicates that this practice may actually increase harms. There is an absence of research exploring youth perspectives on parental approaches to substance use or the approaches youth find helpful in minimizing substance use-related harms.

**Methods:**

This paper draws on interviews with youth aged 13–18 (*N* = 89) conducted within the Researching Adolescent Distress and Resilience (RADAR) study in three communities in British Columbia, Canada. An ethnographic approach was used to explore youth perspectives on mental health and substance use within intersecting family, social, and community contexts. This analysis drew on interview data relating to youth perspectives on parental approaches to substance use. A multisite qualitative analysis (MSQA) was conducted to examine themes within each research site and between all three sites to understand how youth perceive and respond to parental approaches to substance use in different risk environment contexts.

**Results:**

Within each site, youths’ experiences of and perspectives on substance use were shaped by their parents’ approaches, which were in turn situated within local social, geographic, and economic community contexts. Youth descriptions of parental approaches varied by site, though across all sites, youth articulated that the most effective approaches were those that resonated with the realities of their lives. Zero-tolerance approaches were identified as unhelpful and unresponsive, while approaches that were aligned with harm reduction principles were viewed as relevant and supportive.

**Conclusions:**

Youth perspectives illustrate that parental approaches to substance use that are grounded in harm reduction principles resonate with young people’s actual experiences and can support the minimization of harms associated with substance use. Evidence-based guidance is needed that supports parents and young people in adopting more contextually responsive harm reduction approaches to youth substance use.

## Background

The World Health Organization (WHO) has identified youth as a priority population at risk for problematic substance use [1]. Global statistics demonstrate that substance use is concentrated in youth populations [2], and the WHO [1] identifies youth as particularly vulnerable to substance-related harms. In response to these concerns, the United Nations’ General Assembly convened a special session in 2016 focusing on the world’s drug problem, which included a roundtable discussion focusing on the “significant health challenge” of child and youth substance use [3]. Further, this roundtable highlighted concerns affecting this population including substance use stigma, limited accessibility of treatment, and inadequate youth programming utilizing principles of harm reduction.

Recognizing the key role that parents play in influencing the amount and frequency of youth substance use as well as resultant harms, there is a strong focus in research and practice to develop and deliver public health campaigns and family-based interventions aimed at supporting parents in minimizing the harms of youth substance use [4–6]. To date, much of this work has been in the area of drug use prevention, which has largely reflected abstinence-based, zero-tolerance ideologies [7]. However, such approaches have been critiqued as unresponsive to many youth contexts, and ineffective in reducing harms associated with substance use, in part because they deter youth from seeking help in instances of unsafe use, such as intoxicated driving or over-consumption [7–10]. Youth voices have been absent from the development and implementation of these approaches, which has contributed to the lack of resonance of parental strategies with the actual context of youths’ lives, and has limited opportunities for parental responses to youth substance use that effectively minimize harm [10].

Against this backdrop, parents may remain unsure about how to address substance use with their children. Resultantly, the approaches adopted by parents related to youth substance use are frequently ad hoc [11, 12] and not grounded in evidence. One example of this that has been extensively studied is parental provision of substances, specifically alcohol [13]. Parental provision of alcohol is a widely utilized practice, often with the intention of promoting moderation and minimizing substance-related harms [13]. However, data from recent systematic reviews and cohort studies concludes that this practice is associated with greater levels of adolescent alcohol use, heavy drinking, and alcohol-related problems [13, 14]. Yet, there are limited qualitative studies, and particularly limited studies involving youth voices, which could provide the nuance needed to more fully understand the aspects of this practice that contribute to identified harms. Further, what we do know from qualitative work in this area is that there are contextual factors, including family context and place of consumption, that play important roles in producing identified harms [15]. As the abstinence-based campaigns and parental provision literature help to demonstrate, there is a paucity of resources available for families hoping to adopt evidence-based harm reduction strategies [16].

Harm reduction is conceptualized as an approach that focuses on reducing the health, social, and economic harms of substance use without requiring abstinence. Further, a harm reduction approach aims to enhance knowledge of lower-risk use and to address the risk and protective factors associated with harms [17]. In this way, harm reduction can provide a pragmatic strategy for addressing youth substance use, and one that holds relevance across the spectrum of use practices, from abstinence through to problematic use and substance use disorder. Exploration into the impacts of harm reduction-oriented youth substance use programming is beginning to emerge. In the school setting, harm reduction programs have been demonstrated to increase youth engagement and adoption of content, and reduce reported substance-related harms [18–20]. However, harm reduction approaches have yet to be widely applied to parent-targeted youth substance use intervention [16], an opportunity that requires attention.

In developing parent-targeted, harm reduction interventions for youth substance use, it is key to do so while centering youth perspectives—a key element of a harm reduction approach. Youth perspectives on adult approaches to substance use are frequently absent from research and policy, and are crucial to informing strategies that resonate with youths’ lived realities [20]. This manuscript contributes to addressing identified gaps by illuminating youth perspectives on parental approaches to substance use, drawn from the Researching Adolescent Distress and Resilience (RADAR) study. The language of parent is used throughout this paper, but is intended inclusively to refer to parents, guardians, or other adult caregivers. This qualitative study engaged youth from three communities in British Columbia, Canada to explore youth experiences of mental health, substance use, and resilience. Drawing on analysis of youth narratives on their parents’ attitudes and behaviors toward youth substance use, this study was guided by the research question: How do youth variously perceive parental approaches to youth substance use, and what types of parental approaches do youth find helpful in minimizing substance use-related harms?

## Methods

The findings from this analysis of youth perspectives on parent approaches to substance use are drawn from the RADAR study. Conducted in British Columbia (BC), Canada between 2012 and 2014, this multisite qualitative study drew on ethnographic approaches to understand how youth experiences of mental health and substance use are shaped by their family, social, and community contexts [21]. To this end, methods included individual interviews with youth in three communities in BC, as well as participant observation and field notes that facilitated examination of how the context of each research site shaped participants’ experiences [22]. Field notes contributed to the development of researchers’ understanding of the three research sites and to the initial broad coding of interviews; however, interview data is used for this analysis to center youth narratives. This paper’s analysis draws on data from 83 interviews conducted with youth ages 13–18 across the 3 research sites, which are described in detail below. Following, we outline the theoretical framework informing this study, our data collection processes, and our analytic strategy.

### Research sites

The three sites selected for this study represent urban, suburban, and rural geographies and are identified by pseudonyms: The City, The Valley, and The North. In describing the unique features of each site, we aim to illustrate how socio-geographical context shapes the results of our analysis and how youth and their parents perceive and respond to substance use. The City is a major metropolitan center, with an approximate population of 631,000 within the municipality, and over 2.5 million in the larger regional area [23]. The municipal district in which this study was conducted is wealthier overall compared to The City and the rest of the province, and residents have double the rate of higher education than within The City overall [23, 24]. The City is culturally and ethnically diverse, with 48% of people identifying as first-generation immigrants. Fifty percent of the total population is of Asian origins [24].

The Valley is a suburban city, with an approximate population of 140,000 [24]. The immigrant population in The Valley is smaller than in The City, with 27% of the total population having immigrated to Canada. Though statistics regarding race are not measured directly, The Valley is predominantly white, with 61% of the population being of European descent. However, there is also a large South Asian community, which constitutes 31% of the population [24]. Within this community, Punjabi is predominantly the language spoken at home [24]. The Valley is a highly religious municipality overall, with only 27% of the population claiming no religious affiliation, compared to 49% in The City [23]. The two primary religions in The Valley are Christianity (50% of the total population) and Sikhism (20%).

The North is a rural community that encompasses a small town and the surrounding area, as well as First Nations reserve land. The total population of the area is approximately 4800 [25]. The town has experienced a population decline of 5.5% since 2011; in comparison, The City and The Valley have had population growth of 5.9% and 4.6% respectively during this time [24]. In addition to the population of people living on reserve land, 29% of the in-town population identifies as Indigenous compared to 6% nationally [24]. The North, as with many communities in Canada, has been shaped by a long history of colonialism, which has significantly impacted social and health outcomes for Indigenous populations. Indigenous people in Canada continue to experience systemic violence, including through neocolonial policies and inaccessible health systems, and stigmatizing health and social encounters [26]. As a result of this historical and ongoing trauma, many Indigenous people experience social inequity and health disparities [27]. In The North, many people have experienced intergenerational trauma and live in poverty without equal access to social and health resources.

### Theoretical framework

Given our focus on better understanding youth perspectives on parental approaches to substance use, with the broader aim of supporting improved parental strategies to reduce the harms of youth substance use, our analysis is informed by Rhodes’ [28] risk environment framework for harm reduction. This framework critiques individualistic understandings of harm reduction, which position harms resulting from substance use as an issue of individual acceptance or avoidance of risk, and frame harm reduction as primarily a strategy for supporting individual behavioral change [28]. The risk environment framework shifts the positioning of risk from the individual or the notion of a ‘high risk’ social group to the multiple intersecting environments that contextualize a person’s life and experiences of substance use [29]. Rhodes [28] conceptualizes environment as the intersection of social/political, economic, geographical, and policy environments at both the micro-level of immediate and proximal influence on a person’s experiences, and the macro-level of broader conditions that contextualize substance use including inequities in health and social systems. The risk environment framework therefore contributes to this analysis a more holistic and nuanced understanding of factors that may shape young people’s perspectives on substance use and the multitude of environmental factors that influence individuals and families in navigating social narratives of youth substance use. In its focus on context, this framework supports our analysis of youth perspectives on substance use as situated both within family experiences and within broader geographical, social, and political contexts that shape families’ beliefs, attitudes, and values.

### Data collection

This study received ethics approval from the University of British Columbia Behavioral Research Ethics Board, and was granted permission by each research site’s school district to collect data from youth participants. Youth were recruited through posters advertising the study within schools, and with assistance from school and community staff. Specifically, school counseling staff assisted the research team in recruiting youth with diverse experiences and perspectives. Youth provided their own consent to participate, and did not require parental permission. Individual interviews were conducted in private spaces within each school, and covered a wide range of topics related to distress and resilience, including mental health, substance use, family and peer relationships, and school support. Aligned with ethical guidelines, the research team was prepared with information and guidelines for referral to support services if a participant expressed distress or identified risks to safety during the interview. Researchers conducting the interviews all had training in mental health, substance use, and in working with youth, and were all women. Interviews were between 30 and 120 min in length. Participants each received a $20 CAD honorarium in appreciation of their time. Each participant was offered the opportunity to review their transcript for accuracy. Additionally, a community report was developed for each research site, which provided the opportunity for youth to provide feedback on findings and analysis prior to publication.

### Data analysis

All interviews were transcribed and accuracy checked. Thematic analysis was conducted to code interviews [30], resulting in 15 broad codes that reflected the participants’ experiences of distress and resilience, as well as their family, peer, and community contexts. For example, codes included *Friends and Social Connections*, *Family*, *Identity*, *Violence*, *Aspirations and Goals*, and *Coping Strategies*. Codes were not mutually exclusive, to capture the ways in which youth experiences intersected with a wide range of contextual factors. This analysis primarily draws from the *Substance Use* code. Following broad coding, *Substance Use* data were analyzed through a multisite qualitative analysis (MSQA) approach [31]. MSQA is an analytical approach developed through our previous analyses of RADAR data, and aims to elucidate themes both within and between diverse study sites. While many approaches to multisite analysis aggregate findings from all sites, or analyze each site’s data as separate case studies, MSQA facilitates the development of overarching experiences across sites, while preserving contextualized understandings of experiences at each site.

Drawing on the MSQA *within*-*between*-*within* process of analyzing data, we first analyzed youth experiences of substance use separately for each study site to gain in-depth understandings of the contextual features of each site and to generate within-site themes. In the next (between-site) stage, we identified the overarching theme of parents/caregivers shaping youth perspectives on and experiences of substance use. Using this theme as a lens for the third and final (within-site) stage of the process, we returned to each site separately to further develop site-specific findings and to ensure contextual relevancy. In each stage of the MSQA process, analysis was conducted first as an independent process, followed by team discussion of emergent findings to enhance nuance our analyses. In this analysis, we aimed to elucidate how the context of youth’s lives at each site shaped how their families perceived and talked about substance use, and how youth responded to their parents’ approaches. Thus, the MSQA approach facilitated analysis of how young people’s experiences of and perspectives on substance use are shaped by intersecting micro- and macro-levels of Rhodes’ risk environment framework, including family perspectives and the broader community’s social, economic, and geographic contexts.

## Results

A total of 83 youth participated in individual interviews, including 29 in The City, 27 in The Valley, and 27 in The North. Table 1 provides an overview of the demographics of youth participants from each site. Demographic data on ethnicity were determined by participants’ self-report and/or gleaned from participant interviews. The study sample was diverse in the types of substances used, including alcohol, cannabis, mushrooms, and cocaine. Findings from our MSQA are presented by site to illustrate the unique ways in which youth in each setting articulated their parents’ approaches to substance use. For each site, we first describe the predominant parental approach to substance use as described by the young people in this study, then discuss how youth perceived and responded to this approach in managing their own use.

**Table 1:** Participant demographics (*N* = 89)

	The City		The Valley		The North		Totals	
	*N*	%	*N*	%	*N*	%	*N*	%
Gender								
Female	14	48%	12	44%	14	52%	40	48%
Male	13	45%	15	56%	13	48%	41	49%
Non-binary	1	3%	–		–		1	1%
Left blank	1	3%	–		–		1	1%
Age								
13–14	10	34%	–		4	15%	14	17%
15–16	11	38%	12	44%	11	41%	34	41%
17–18	8	28%	15	56%	7	26%	30	36%
Not provided	–		–		5	19%	5	6%
Ethnicity								
White/European	16	55%	11	41%	4	15%	31	37%
Multi-racial	5	17%	2	7%	4	15%	11	13%
Indigenous	–		–		18	67%	18	22%
Asian	3	10%	1	4%	–		4	5%
South Asian	–		12	44%	–		12	14%
West Asian	1	3%	–		–		1	1%
Latin American	1	3%	–		–		1	1i
Black	–		1	4%	–		1	1%
Unknown	3	10%	–		1	4%	4	5%

### The City: using within established limits

In The City, participants predominantly described a family context in which their parents and caregivers accepted their substance use, but within defined limits. For one participant, these limits related to both age and degree of alcohol consumption: “My 19-year-old sister, so with her it’s like they accept that she drinks. [If] she gets drunk though, she’ll get in trouble like, they’re gonna give her the talking-to … but she’s over-age” (19 is the legal drinking age in BC). While in this family, drinking was acceptable over the legal age limit, there was also an understanding that alcohol should be consumed in moderation. Other participants described a similar approach of parents and caregivers permitting moderate alcohol consumption, such as serving one drink with dinner, or allowing their child to get “a bit tipsy” at a special family gathering. In one participant’s words:

I mean like my parents, they did a good job, they did not say “okay, no alcohol for you at all” they kinda like they started me off with it, like “you can have a glass of wine with dinner”. So occasionally, after work on the weekend, I would have like a long day in the sun, in the sun, right? Kind of working outside, so like, “here, sit down I’ll get you beer” right? So we have a beer and watch the hockey game.

Similarly, another participant stated, “I usually drink wine with my parents”; while small amounts of wine are normalized within the family, she learned from her parents that “we have limits.” Youth described this approach of permitting use while encouraging moderation as “relaxed,” and articulated that families that acknowledged that some substance use would occur had more realistic and reasonable expectations of young people.

Participants whose families permitted use within limits took up these limits in their own approaches to substance use, endorsing small amounts of alcohol consumption and often resisting consuming alcohol to a degree that would result in becoming drunk, which was viewed as excessive. One participant described, “I don’t really have the need to go out and just get piss drunk … it’s not like ‘okay I just wanna drink just for the purpose of getting wasted.’ I don’t have that.” Having learned at home that small amounts of alcohol could be enjoyable without negative consequences, this young person adopted an approach of moderation when socializing with peers. Similarly, another participant stated, “when you’re a kid you drink a bit of wine and, sure, you get drunk sometimes because you have a family party, you drink the rest of the champagne, like you’re sort of a bit tipsy, it’s kind of fun.” She contrasted this approach of moderation with “you go to a party, you see girls that are smashed.” Extending from her own family’s approach of encouraging moderation, she viewed getting excessively drunk at parties with peers as undesirable alcohol consumption, while getting “tipsy” in a safe setting was enjoyable. For many young people in The City, encouraging moderation was viewed as a more effective family approach to substance use than permitting use without limits. One participant noted that in the absence of parental limit-setting, youth may not develop strategies for moderating their own use: “the parents don’t care, the parents think it’s fine … and the kids just don’t learn.” This form of leniency was viewed by participants as ineffective for supporting youth in developing strategies for self-management of substance use, while supporting use within limits was consistently viewed by youth as an effective parental strategy for encouraging moderation.

### The Valley: responding to zero-tolerance

In the Valley, participants’ families predominantly addressed substance use through a zero-tolerance or abstinence-based approach, with youth recounting explicit expectations from their parents that their children avoid all substance use. Messaging that substance use is unacceptable under any circumstance or in any amount was common within youth descriptions of their families. For example, one participant stated that her mother consistently told her and her siblings “that we better not do any of *that* … we’ve been told this since we’re very young, like grade 5 probably.” While for many families zero-tolerance was an overt expectation, some youth also described family contexts in which abstinence from all substances was assumed but not discussed. One participant recounted a period in her earlier teenage years when she went “down the rough road” of drinking alcohol and using substances. She described the response of her family as: “my mom didn’t really know what was happening and neither did my dad, but my sister did and she tried telling my mom—but mom’s like ‘oh no, [name of participant] would never do that.’” This lack of awareness about the possibility of youth substance use was also reflected in how some participants perceived their peers’ families. As one participant speculated, some parents “they’re kind of oblivious to the fact that ‘my kid’ could actually do something like that.” Zero-tolerance approaches were frequently described as a disconnect from the realities of young people’s substance use.

For some youth, a zero-tolerance approach in their families resulted in their taking up of abstinence from substances. As one participant described, “I’ve been offered a lot of times, I’ve been tempted to, but I always think that you know, I can’t cause my parents don’t, and they expect me not to.” The participant who received early zero-tolerance messaging from her mother similarly stated: “the time came when people started doing these things around grade 8—we didn’t have to think about it. We already knew that that’s not something that we were going to do.” However, other participants whose parents and caregivers adopted a zero-tolerance approach used substances despite their family’s expectations and experienced negative effects on their family relationships and difficulties in managing their own use. One participant who reported occasionally drinking alcohol in moderation described the impact on her relationship with her mother: “when she was a teenager she never did any of that, she never drank, she never did any drugs … she never did anything bad. So, to her, that’s like, I’m going to hell.” Another participant who used substances described that her grandfather “was starting to like, disown me—he didn’t want to be around me” before she became abstinent. In addition to impacting family relationships, a zero-tolerance approach was also described as leaving youth without strategies for managing their own use or talking to their peers about substances. One participant shared his distress about a friend’s heavy cannabis use, which he perceived as problematic, and stated that his friend’s parents were aware of and permitted his use. This participant’s own family supported a zero-tolerance approach, which limited his development of strategies for discussing his concerns with his friend. He stated, “I just can’t help him now, ‘cause his parents won’t help him right?’ … I just can’t help him if his dad’s not going to say anything.” For youth in The Valley, zero-tolerance family contexts did at times result in abstinence. However, for both youth who used and did not use substances, a zero-tolerance approach was described as negatively impacting relationships, preventing open dialog about substances, and conveying the message that young people could not be trusted by adults.

### The North: navigating intergenerational substance use

In The North, youth described substance use as visibly commonplace in town and frequent among the adults in their live. Reflective of Rhodes’ risk environment, participants frequently spoke to how this community context shaped both their families’ and their own perspectives on substance use. While substance use was not universal, and the type and frequency varied, participants’ descriptions of their community consistently highlighted the prevalence of substance use. One participant stated, “there’s a lot more drinking and everything here, just cause it’s so small”, while another characterized the town as “pretty good … until you have some people into drugs and that, trying to go, ‘hey, have something.’” In this community context, most participants described having family members—including parents—who used substances or drank heavily. One participant described her mother as having been in treatment for substance use, while another stated that her father’s “drinking a lot last year” led to “problems” for the rest of the family. While some of the youth discussed their experience with a parent or caregiver who used substances, in other families, substance use and drinking were widespread. A young woman in The North described a period of time when her mother “got into drugs … it kind of got the best of her”, while another stated, “my grandfather’s an alcoholic, my grandmother, my grandfather, my step-grandma, are alcoholics.” Unique to The North, participants also described attending parties that were intergenerational, whereas in the other research sites substance use occurred within peer groups and often without adults’ awareness. One participant described these parties as “mixed” between youth and adults, with “people drinking all over on weekends and a lot of noise.”

Many participants described their parents’ substance use, previous or ongoing, as shaping the family context for substance use and impacting their own opinions and use. Some participants held the belief that parental substance use would inevitably lead to problematic use in children—“if their parents are alcoholics or they do drugs or anything, they don’t really have the strength to say no.” However, other participants challenged this narrative, describing how experiencing their parents’ use contributed to their own negative view of substance use. One participant whose father previously used substances stated, “I don’t do that stuff because my dad told me that he went to jail that one day when he did it once, then he told me not to do it … told me not to drink or anything.” Another participant who described her mother as “an alcoholic” stated that people “think I’ll turn out like my mom and stuff. They think I’ll be like her, drink and do drugs, and be a troublemaker too and stuff, but I’m nothing like her.” While this individual drank regularly at parties with friends, she differentiated her use from her mother’s in that she is avoiding “addiction” and pursuing life goals—“trying to finish school so I won’t have to be like her.” Due to the ubiquitous nature of substance use in the community, including among some of the participants’ family members, many families attempted to navigate their children’s use by encouraging open communication and responsible use, demonstrating complex intersections between Rhodes’ micro- and macro-level risk environments. For example, one participant stated that her mother will obtain alcohol for her “as long as I’m responsible.” Another participant described her father’s approach to her substance use as:

“you can’t chain a dog to a porch”, so he’s saying that it’s like a metaphor like, you cannot stop kids from doing what they do when they are out with their friends, like you have no idea what they could do. And so many parents do not know anything about what their children do, and with my dad, because we are so close he knows what I do and he trusts me a lot.

In many families, parents acknowledged youth substance use, but encouraged moderation. One individual’s mother “let me have a few sips out of her drink, or she’ll mix me a really weak one”, and another participant’s father “told me to drink only on special occasions.” Each of these participants took seriously and maintained these limits established by their parents in their own alcohol consumption. For others, witnessing negative effects of substance use in their families led to independently developing limits for their own use: for example, one participant described using cannabis exclusively and avoiding all other substances, stating “I see what that stuff does.”

### Between-site youth perspectives on substance use

Across all three sites, youth articulated that family approaches to substance use were most effective when they resonated with the realities of young people’s lives. Many of the youth described their parents as having a lack of understanding or an inability to relate to the contexts of substance use for youth, which they identified as not helpful for supporting youth in managing their own use. One participant from The North reflected,

Once they become an adult and have a family and stuff, something switches and then they do not understand this side. And they are kind of, like, parent view. They do not quite understand things. They do not see it from our point of view, and they kind of think of it as nonsense or they just do not see the full perspective. They just see you, but they do not see what’s happening and what’s around you and what’s in you … Very straightforward perspective of their kids, I guess.

As a result, many youth across the research sites avoided discussing substance use with their parents out of a concern of “worrying” them, and suggested that any conversation about substance use was taboo within the family. As stated by one participant from The Valley: “my mom always gets upset every time I talk to her about this right?”. Conversely, youth reflected that parents being too “lenient” about substance use was also unhelpful, and did not support youth to use within limits. One participant from The North who described drinking alcohol frequently stated she was “sick of it” but found it difficult to reduce her consumption, as her parents “don’t really care about what I do. I could go home drunk and they won’t do anything.”

Participants between the three research sites articulated that both unrestricted leniency and zero-tolerance approaches to substance use are unhelpful and do not resonate with actual contexts of substance use in youths’ lives. Rather, youth reflected that openly discussing substance use within the family and parental modeling of use in moderation are responsive approaches to youths’ actual use and helpful in managing potential harms associated with substance use. Many participants called for open communication with their parents about drug and alcohol use “without being afraid of the consequences”. Across sites, participants articulated that they value honesty and trust, and reflected that the most helpful family approaches are those that seek to understand and respond to youths’ existing perspectives on substance use.

## Discussion

Parents are frequently held responsible in society for shaping their children’s perspectives on substance use; however, there is limited guidance for parents in effectively communicating with their children, or promoting substance use behaviors that minimize harm. Additionally, current literature on parental approaches to youth substance use limitedly draws on the lived experiences of young people to inform understandings of what approaches may be helpful, and how youth respond to parental messaging about substance use. To support parents in more effectively engaging with their children about substance use, evidence-based approaches are needed that are responsive to the realities of youths’ lives and support them in understanding and reducing harms of substance use—without drawing on ineffectual “scare tactics.” This paper draws on youth perspectives to further develop nuanced understandings of youth substance use as situated in complex risk environments [28], encompassing both family approaches and broader geographical and socio-political contexts. Further, our findings illustrate that parental zero-tolerance approaches do not resonate with the realities of youths’ experiences of navigating substance use, and that across different community contexts, harm reduction approaches are more responsive to youth perspectives and needs. Within harm reduction philosophy, lived experience is foundational to understanding “what works” and informing policy and programming; yet, youth perspectives are frequently absent from discourses on parental approaches to youth substance use [20, 32]. This paper presents qualitative insights into youth responses to parental attitudes toward substance use, including the practice of parental provision, which requires more nuance in the current literature. Findings from this analysis focused predominantly on parental provision predominantly related to alcohol. This may firstly reflect that alcohol is the most likely substance for parents to be using—particularly openly, with their children’s knowledge. Additionally, participants may have selected to highlight these stories within the interview context given that, at the time of interview, alcohol was the only legal substance described. However, while narratives of parental provision centered predominantly on alcohol, young people’s perspectives on parental approaches to substance use reflected attitudes to a wide range of substances.

In social discourse, youth who use substances are often positioned as deviant “bad kids” who reject all authority, including that of their parents [33]. This framing has been thoroughly criticized in the research literature, which has increasingly sought to understand the context for youth decision-making around substance use. However, in seeking underlying factors that contribute to youth substance use, the locus of blame has shifted toward parents. Research studies have frequently examined the role of parents in contributing to their children’s substance use, and cite factors such as the parent-child relationship, parenting style, and poor role modeling as having potentially negative impacts on youth substance use [34, 35]. This study’s analysis challenges such narratives of blame that position either youth or their parents as “at fault” for youth substance use. Rather, our findings support a “risk environment” understanding of both the broad and local contextual factors that shape youth experiences of substance use [36]. At the environmental micro-level, participants’ family contexts and parental messaging impacted their attitudes and behavior regarding substance use and management of potential substance-related harms. Additionally, at the macro-level, broader societal, political, economic, and geographical factors contextualize families’ beliefs, values, and perspectives. For example, in The North, historical and current colonization has produced a community context in which substance use as a trauma response is widespread, and many youth reported having family members who experienced problematic substance use [37, 38]. In The Valley, the prevalence of religion has contributed to a more socially conservative population overall, perhaps explaining the predominantly zero-tolerance parental response to youth substance use in the families of participants we interviewed [39, 40]. This macro-level analysis of the context for youth substance use is frequently missing from the research literature, which risks perpetuating narratives of blame surrounding parental approaches and fails to capture key factors that shape youth perspectives and experiences. Programming and policy that aim to address youth substance use or parental messaging on substance use must be responsive to not only the micro-level family context but also the broader macro-level factors that families are variously situated in and impacted by. In this way, a risk environment lens supports an understanding of youth substance use as informed by parental messaging and practices, without reproducing discourses of blame.

While harm reduction has emerged as a predominant approach for addressing substance use in adult populations and is beginning to be used as a guiding philosophy for school-based substance use programming, messages to parents about addressing their children’s substance use remain largely grounded in zero-tolerance. Governmental messaging campaigns and policy approaches to youth substance use frequently seek non-controversial approaches, and therefore advance abstinence-based approaches as the best or only condoned parental strategy [7]. For example, the government-led National Institute on Drug Abuse [41] in the USA centers its messaging for parents on “preventing teen drug use” and reinforces the notion that “positive parenting prevents drug use”. Similarly, the Government of Canada’s [42] advice to parents cautions that young people “may be using drugs and might need help to stop”. Our findings on youth perspectives on family approaches to substance use illustrate that across all three geographical sites, prevention of all substance use was not an effective message for youth. In The City and The North, zero-tolerance was not representative of the reality of parental attitudes, which tended toward supporting moderation in use. In The Valley, where zero-tolerance was a common parental approach, participants reflected that parents’ lack of understanding of or communication about substance use was unhelpful and their expectations of abstinence unrealistic. Abstinence-centered messaging for parents may resonate with the zero-tolerance approach of some families, such as in the more religious and conservative Valley. However, this approach fails to prepare or provide direction for parents in supporting youth in learning to moderate their own substance use, and reduce associated harms. In contrast, across all three sites, harm reduction approaches consistently resonated with youth, who described integrating their parents’ messaging of using within defined limits and balancing use with harm minimization.

A potential limitation of this study is that as recruitment was conducted through schools at each research site, youth with poor attendance (or who are not currently enrolled in school) may not be represented in the study sample. Additionally, youth participants may have experienced discomfort in talking to adults about substance use, though this may have been mitigated by researchers’ experience building rapport with young people. Overall, members of the research team found youth participants to be candid and open in sharing their experiences across many difficult topics, including substance use, and experiences of racism and bullying [43]. Despite these limitations, a strength of this study is the presentation of findings from across a diverse set of communities, each with unique contexts that shape youth experiences of substance use and perspectives on parental approaches to substance use. The MSQA approach of analyzing data within and between sites contributes to findings with broader applicability across diverse settings and populations. Findings from this study illustrate that in talking to their children about substance use, parents are drawing on approaches that align with harm reduction messaging and that these messages resonate with youth experiences and perspectives across diverse communities. Programming, policy, and media campaigns that advance zero-tolerance as the only acceptable parental approach are unlikely to resonate with all parents, and risk leaving parents and their children with little guidance for minimizing harms within the context of some use [44].

## Conclusions

Zero-tolerance continues to be promoted as the most socially acceptable strategy for parents in talking to their children about substance use. However, our findings illustrate that abstinence-only messages do not resonate with the realities of youth experiences or perspectives on using substances, and suggest that parental messages that support youth in using within limits and in building capacity to minimize harm may be more resonant and impactful. In addition, while harm reduction messaging may be more responsive to youth experiences across diverse geographical and social contexts, parents continue to have limited guidance or support in drawing on harm reduction principles in communicating with their children. Despite a social and political climate in North America and many other Western countries that upholds zero-tolerance as ideal, findings from this study contribute to the body of literature illustrating that evidence-based messaging, programming, and policy are needed that support parents in developing the necessary knowledge and capacity to integrate harm reduction principles into family responses to youth substance use.

## Abbreviations

CAD: Canadian dollar
MSQA: Multisite qualitative analysis
RADAR: Researching Adolescent Distress and Resilience
WHO: World Health Organization
